# Comparative Study between Curcumin and Nanocurcumin Loaded PLGA on Colon Carcinogenesis Induced Mice

**DOI:** 10.3390/nano12030324

**Published:** 2022-01-20

**Authors:** Farida E. Elbassiouni, Wafaa M. El-Kholy, El-Sayed M. Elhabibi, Sarah Albogami, Eman Fayad

**Affiliations:** 1 Department of Zoology, Faculty of Science, Mansoura University, Mansoura 35516, Egypt; dr_wafaa_s@mans.edu.eg (W.M.E.-K.); e.esmail@tu.edu.sa (E.-S.M.E.); 2 Department of Biotechnology, Faculty of Sciences, Taif University, P.O. Box 11099, Taif 21944, Saudi Arabia; dr.sarah@tu.edu.sa

**Keywords:** colorectal cancer, inflammatory factors, VEGF, DMH and curcumin encapsulated PLGA

## Abstract

Colorectal cancer is the third most common cancer. Because curcumin (CUR) has anti-inflammatory and anticancer properties, research has been undertaken to indicate that nanocurcumin compounds can be used to treat a variety of cancers. CUR in nanoform has been found to have a stronger effect than conventional CUR. The purpose of this study was to show that CUR-loaded poly lactic-co-glycolic acid nanoparticles (PLGA) (CUR-loaded PLGA) have anti-inflammatory and anticancer effects on colon carcinogenesis in male dimethyl hydrazine (DMH) mice as a comparative study between the nanoform of curcumin and normal curcumin, focusing on the anticancer effect of nanocurcumin. Mice were separated into six groups: No treatment was given to Group I (negative Group-I). Group II was treated with CUR. Group III was treated with CUR-loaded PLGA. Group IV was treated with DMH. Group V received DMH and curcumin. Group VI received DMH and CUR-loaded PLGA. At the conclusion of the trial, the animals were slain (6 weeks). Inflammatory indicators and vascular endothelial growth factor (VEGF) levels all changed significantly in this study, as the following inflammatory markers as TNF showed percent of change compared to the DMH group. Recovery percentage for Groups V and VI, respectively, were 9.18 and 55.31%. In addition, IL1 was 7.45 and 50.37% for Groups V and VI, respectively. The results of IL6 were 4.86 and 25.79% for Groups V and VI, respectively. The vascular endothelial growth factor (VEGF) recovery percent was 16.98 and 45.12% for Groups V and VI, respectively. Following the effect of DMH on colon mucosa shape, the researchers looked at the effect of CUR-loaded PLGA on colon histology. It was shown that CUR-loaded PLGA affects the cell cycle and PCNA expression. We conclude that nanocurcumin is an important anti-inflammatory and cancer-fighting agent.

## 1. Introduction

Cancer is the result of the uncontrolled proliferation of cells of any type, so there are several kinds of cancer that are different in their response to treatment and also in their behavior [1]. Colorectal cancer (CRC), commonly known as big bowel cancer, is characterized by the storage, fermentation, absorption, secretion, and motility of the colon. The junction of the rectum and the rectosigmoid CRC is a global public health concern. In 2018, 1.8 million people worldwide were diagnosed with colorectal cancer, indicating that more research is needed [2]. CRC begins as polyps, or noncancerous expansions of mucosal epithelial cells, and proceeds slowly for 10–20 years before becoming malignant. The granular cells develop into an adenoma or polyp in the large intestine, which generates mucus [3].

Phytochemicals are the second metabolites of plants that are not necessary for growth but have a great effect on human health. They may also play a role in the defense of plants against environmental conditions. Phytochemicals have many effects, such as acting as anticancer, anti-inflammatory, or antimicrobial agent. Phytochemicals are very useful for humans in their native form or as metabolites. CUR is one of the phytochemicals that, in nanoform or encapsulated in nanoparticles, enhances the absorption and bioefficacy of CUR [4]. The removal of damaged organelles inside the cell is called autophagy. CUR acts as an autophagy producer and was used in the treatment of thyroid cancer [5]. Autophagy inducers such as CUR are also used in the case of chemotherapeutic resistance in patients with colorectal cancer. CUR is used in resistance cases because it is able to induce apoptosis in cancer cells that show apoptotic resistance [6]. Cancer cells have the ability to resist chemotherapy as it makes DNA repairable after treatment with chemicals or drugs that damage the DNA structure, another type of resistance [7]. CUR can also reduce the side effects of chemotherapy by reducing the levels of the reactive oxygen species (ROS) from the chemotherapy [7]. Chemotherapy in cancer causes organ toxicity such as hepatotoxicity, cardiotoxicity, and renal toxicity as well as many other side effects. When cisplatin is used as chemotherapy, all of these side effects can be reduced by using CUR or its derivatives [8].

CUR contains a wide range of characteristics, including analgesic, antiseptic, anti-inflammatory, antioxidant, insect repellant, antimalarial, and more when used in nanoform. CUR also possesses anticancer and antioxidant effects [9]. CUR has anti-inflammatory benefits. TNF, IL-1, IL-6, and chemokines are examples of inflammatory cytokines. TNF is suppressed, which is one of the most pro-inflammatory cytokines. CUR inhibits the synthesis of pro-inflammatory enzymes [10]. CUR lowers the quantity and activity of EGF receptors (EGFR) as well as the expression of vascular endothelial growth factors (VEGF) to limit the role of epidermal growth factor (EGF) [9]. CUR is a natural anticancer molecule that causes cell cycle arrest and has been extensively studied in the literature. CUR caused cell cycle arrest in the G0/G1 or G2/M stages [11].

During replication, PCNA assists the polymerase enzyme in building a tight connection with the template DNA, avoiding dissociation. A DNA synthesis step, such as pol, is required for all DNA damage repair metabolic pathways, and PCNA plays a role in these processes [12].

PCNA, which plays a vital role in the cell cycle, finishes the activities of DNA replication and DNA synthesis that occurred in the S-phase of the cell cycle. Drug delivery systems have a wide range of applications, including the use of nanosized biodegradable polymers. Numerous polymers have shown significant results when used as drug delivery systems that carry the drug to a target location, enhancing the therapeutic effect while reducing side effects [13]. For the curcumin delivery, there are many types of nanomethods to enhance the CUR stability and delivery to the cells. These delivery systems such as polymeric nanoparticles are very small and biocompatible and have the ability to circulate in the blood; they include liposomes, nanoparticles, solid lipid nanoparticles, magnetic nanoparticles, albumin, gold nanoparticles, conjugates, cyclodextrins (CD), solid dispersions, micelles, nanospheres and microcapsules, nanogels, nanodisks, metallo-complexes, and many other forms [14].

CUR works against a variety of cancers, including gastrointestinal cancers that include cancers of the oral cavity and salivary glands, esophageal cancer, stomach cancer, and intestinal cancer; hepatic cancer; pancreatic cancer; head and neck cancer; brain cancer; breast cancer; colorectal cancer; and prostate cancer. CUR showed the ability to fight against various types of leukemia such as acute lymphoblastic leukemia, acute myeloid leukemia, chronic lymphocytic leukemia, and chronic myeloid leukemia [14].

Many new applications of nanocurcumin are metal-based, particularly cisplatin and other metal complexes used as anticancer drugs to reduce the toxicity of platinum [15]. Ruthenium, the chemical element with the symbol Ru and a transition metal belonging to the platinum group, when it complexes with CUR, has potential as an anticancer treatment [16]. Another application uses premix membrane emulsification to prepare nanocurcumin, and it is used in the formation of nanoemulsions that are nontoxic and safe for consumption [17].

Another type of CUR nanoform is the formation of CUR with cyclodextrin and cellulose in the form of nanocrystals. This complex showed an antiproliferative effect against CRC cell lines more than CUR alone [18]. In addition, there is a new formulation of CUR involving the immobilization of it with polysaccharide particles to enhance its stability and bioavailability. Ionic cross-linking agents such as chitosan help in the formation of a new complex that is a hydrogel microparticle. The release of curcumin in the colon improved [19].

A Pickering emulsion is an emulsion that is stabilized by solid particles and established to be a CUR delivery system. This method of formation enhanced the bioaccessibility of curcumin and β-carotene and increased their adsorption by the small intestine [20]. Using curcumin-loaded tetrahedral framework nucleic acids is a new strategy in the CUR delivery system that is based on DNA nanostructure. It shows high water solubility and excellent drug stability [21]. Due to the nanosize of PLGA nanoparticles, curcumin and the biodegradable properties of both components have the advantages of nanoparticles and improved tissue and cell absorption ability [22].

## 2. Materials and Methods

### 2.1. Chemicals

Curcumin (M.wt = 24,000), (95% purity), was purchased from Sigma-Aldrich (Saint Louis, MO, USA). Poly lactic-co-glycolic acid (PLGA): (M.wt = 38,000), (50:50 lactide-glycolide ratio; inherent viscosity 1.32 dL/g at 30 °C) was purchased from Sigma-Aldrich Co. (Saint Louis, MO, USA). Poly vinyl alcohol (PVA): (M.W. 30,000–70,000) was purchased from Sigma-Aldrich Co. (Saint Louis, MO, USA). All organic solvents were of HPLC grade (dichloromethane).

1,2-Dimethyl hydrazine (DMH): (purity 99.9%) was obtained from Sigma-Aldrich Co. (Saint Louis, MO, USA). All other reagents were of analytical grade and purchased from local suppliers.

### 2.2. Colon Cancer Induction Protocol

DMH was dissolved in 0.9% saline. The dose of (10 mg/kg bw) DMH powder dissolved in saline 0.2 mL. Animals were given a weekly subcutaneous (SC) injection of DMH over the shoulder into the loose skin over the neck for 3 weeks [23].

### 2.3. Curcumin Encapsulated PLGA Preparation

Curcumin-loaded PLGA nanoparticles were prepared by the emulsion technique of solid-in-oil-in-water (s/o/w) [24]. Characterization of CUR-loaded PLGA, encapsulation efficiency, and particle size with TEM and FTIR were performed according to measurements on previous studies [24].

### 2.4. Animals and Housing

Sixty male albino mice, each with a body weight of 15-20 g, were obtained from the Egyptian Institute for Serological and Vaccine Production, Helwan, Egypt, and placed in specific cages in a room with good air (25 ± 1 °C with 55 ± 5% humidity) and a 12 h light/dark cycle at the animal house of the faculty of science at Mansoura University. Animals were acclimatized for one week with a normal laboratory diet and water ad libitum. All experimental procedures were run under the standard guidelines for care and use of laboratory animals as approved by Mansoura University, faculty of science, and the department of zoology ethics committee’s directives on the use of experimental animals for research.

#### 2.4.1. Animal Groups and Mode of Treatment

After one week of acclimatization, the mice were divided into 6 groups as follows:

Group I: (negative Group I) received no treatment.

Group II: The mice received CUR orally (10 mg/kg bw) six times a week for six consecutive weeks [25].

Group III: The mice received CUR-loaded PLGA orally (10 mg/kg bw) six times a week for 6 consecutive weeks [26].

Group IV: The mice were injected by DMH SC with 10mg/kg in 0.2 saline once a week for three consecutive weeks [23].

Group V: The mice received DMH administered the same as Group IV and received CUR the same as Group II.

Group VI: The mice received DMH administered the same as Group IV and received CUR-loaded PLGA the same as Group III.

#### 2.4.2. Body Weight Changes

The mean average body weight of the animals in each group was calculated each week from the starting point until the end of the experiment (6 weeks).

### 2.5. Samples Collection and Tissue Preparation

#### 2.5.1. Blood Sampling

At the end of the experiment (6 weeks), mice, with overnight fasting, were anaesthetized with diethyl ether (Sigma-Aldrich, Saint Louis, MO, USA). Blood specimens were obtained in non-heparinized glass tubes from the bleeding of retro orbital of each mouse. Serum was separated by centrifugation at 3000 rpm for 15 min and stored at −20 °C.

#### 2.5.2. Determination of Cell Cycle

One milliliter of ice-cold absolute alcohol (Sigma-Aldrich, Saint Louis, MO, USA) was used to fix cells for each tube and kept at +4 °C until using.

Samples were again centrifuged, and excessive ethanol was removed after at least 12 h of fixation [27]. Two hundred microliters of the suspension of cells in citrate buffer was put in a 15 mL tubed Falcon comp (2095). The solution of propidium iodide was protected against light with tinfoil during preparation, storage, and the staining procedure. The solution was mixed, and the sample was filtered through a 30 Mm pore diameter nylon mesh filter to eliminate nuclear clumps in another 5 mL tube (12 × 75 mm, cat. no. 2095, falcon comp). Propidium iodide was added to the samples and run in the flow cytometer within 1 h.

Data analysis was conducted using DNA analysis program MODFIT (Verity Software House, Inc. P.O. Box 247, Topsham, ME 04086 USA, version: 2.0 power Mac with 131,072 KB; Registration No: 42000960827-16193213; Date made: 16 September 1996). This software measured DNA ploidy (diploid cycle percentage and aneuploid cycle percentage) and cell cycle analysis, which calculated the percentage of cells in each phase (G0/G1, S and G2/M) of the DNA cell cycle for each sample.

### 2.6. Hematoxylin and Eosin Stains

The histopathology was carried out according to Bancroft, using the hematoxylin and eosin staining technique [28].

### 2.7. Immunohistochemical Detection of PCNA Polyclonal Antibody

The blocks of formalin-fixed, paraffin-embedded tissue were cut into 4 μm sections and mounted on microscopic slides coated with poly-L-lysine [29]. The sections were dewaxed by using xylene and rehydrated by using various concentrations of ethanol to water. Then the samples were replaced in sodium citrate buffer (10 mM, pH 6.0) for antigen retrieval. The slides were laid out for cooling for 15 min, and then each slide was washed with tris-buffered saline (TBS) for 5 min to decrease the activity of endogenous peroxidase. Slides were then subjected to Ultra block (UltraVision plus Detection System, Thermo Scientific, Fremont, CA, USA) for 10 min to block non-specific bindings [29].

The slides were washed with TBS, incubated at 4 °C overnight with primary antibody PCNA Rabbit polycloned antibody (Product. No. SAB2108448, Sigma Aldrich, Taufkirchen, Germany) at a dilution of 1:500 inside a humidified chamber, and then were washed in TBS again. Next, samples were incubated for 20 min in biotinylated goat anti-polyvalent secondary antibody (UltraVision plus Detection System, Thermo Scientific, Fremont, CA, USA) and then rinsed in TBS. Then the same step of incubation and washing was repeated, but the incubation this time was made in streptavidin peroxidase plus (UltraVision plus Detection System, Thermo Scientific, Fremont, CA, USA) for 30 min. After TBS washing, the final step started by adding the sections on 3,3′-diaminobenzidine (DAB) solution (UltraVision plus Detection System, Thermo Scientific, Fremont, CA, USA) until the sections became brown. The counterstain was Mayer’s hematoxylin. The sections were mounted using mounting media and then visualized under the light microscope [29].

### 2.8. Determination of Inflammation Markers and VEGF

Determination of TNF α, IL-1 beta, IL-6, and VEGF in serum occurred quantitatively by using a monoclonal antibody specific for mouse TNF-α, IL-1 beta, IL-6, and VEGF, and this ELISA kit was obtained from ALPCO (Salem, NH, USA). The intensity of the color measured at 450 nm was dependent on the amount of TNF-α, IL-1 beta, IL-6, and VEGF present in the sample. The concentration level of TNF-α, IL-1 beta, IL-6, and VEGF in serum is expressed as pg/mL [30,31,32].

### 2.9. Statistical Analysis

The results are expressed as mean ± standard error (SE). One-way analysis of variance (ANOVA) was the test used to analyze the data followed by the test of least significant difference (LSD). Statistical analysis was performed with SPSS version 19.00 software (SPSS, Chicago, IL, USA). *p* ≤ 0.05 was considered statistically significant [33].

## 3. Results

### 3.1. Body Weight Change

**Figure 1:**
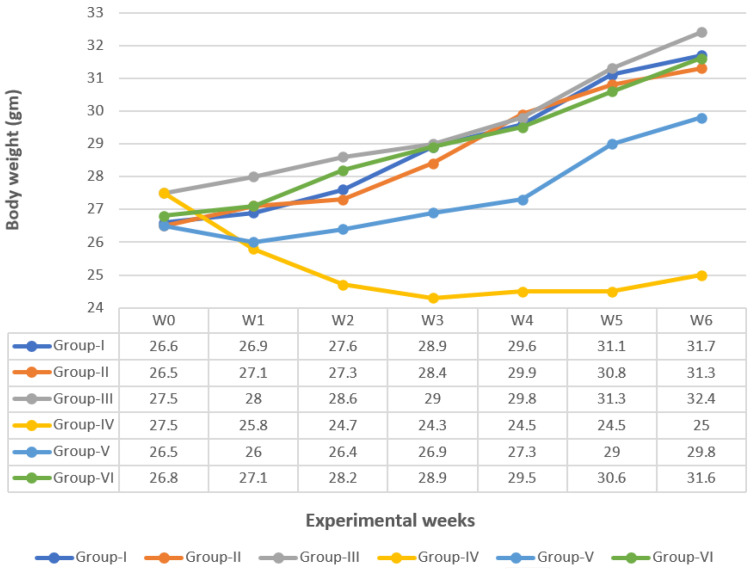
Changes in body weights of different groups under study.

The results showed that the treatment of normal mice with CUR or CUR-loaded PLGA caused non-significant changes in the mean body weight when compared with Group I, Figure 1. The administration of DMH in Group IV afforded a significant decrease (*p* ≤ 0.05) in body weight from the 2nd week until the end of the experiment when compared with Group I. However, co-administration of CUR with DMH in Group V increased body weight significantly when compared with Group IV, although it showed a significant decrease compared with Group I. On the other hand, co-administration of CUR-loaded PLGA with DMH in Group VI increased body weight significantly when compared with Group IV and showed a nonsignificant change when compared with Group I (complete recovery).

### 3.2. Inflammatory Markers and VEGF

**Figure 2:**
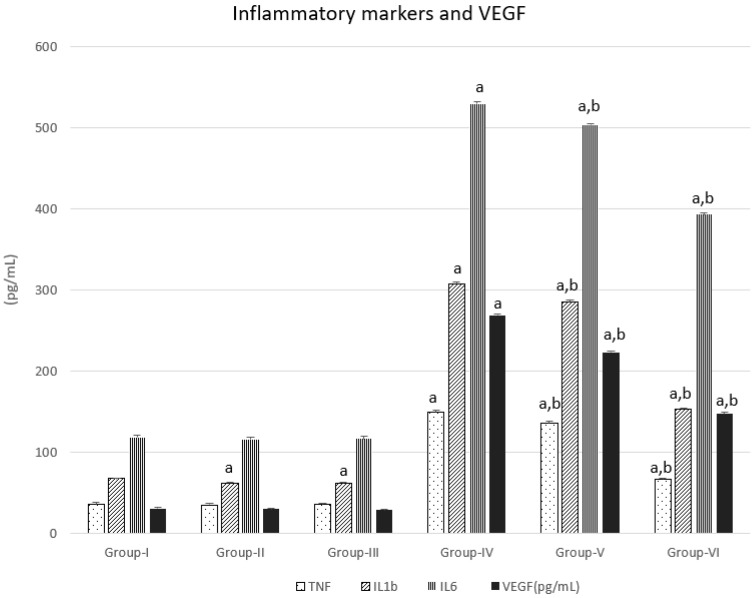
DMH, CUR, and CUR-loaded PLGA effect on serum inflammatory markers and VEGF in the animal groups. a represents statically significant differences at (*p* < 0.05), compared different groups with Group-I and b represents statically significant differences at (*p* < 0.05), compared different groups with Group-IV.

There was no significant change in Groups II and III in TNFα, IL6, and VEGF levels when compared with Group I, except that the level of colon inflammatory markers IL1b significantly decreased in Groups II and III compared with Group I. DMH administration caused significant elevation of serum TNFα, IL1, IL6, and VEGF levels when compared with Group I. Groups V and VI showed significantly improved (reduced) levels of inflammatory markers and VEGF, but they were still significantly higher than untreated Group I. The greatest improvement (decrease) in inflammatory markers was shown in Group VI, Figure 2.

### 3.3. Cell Cycle Results

**Figure 3:**
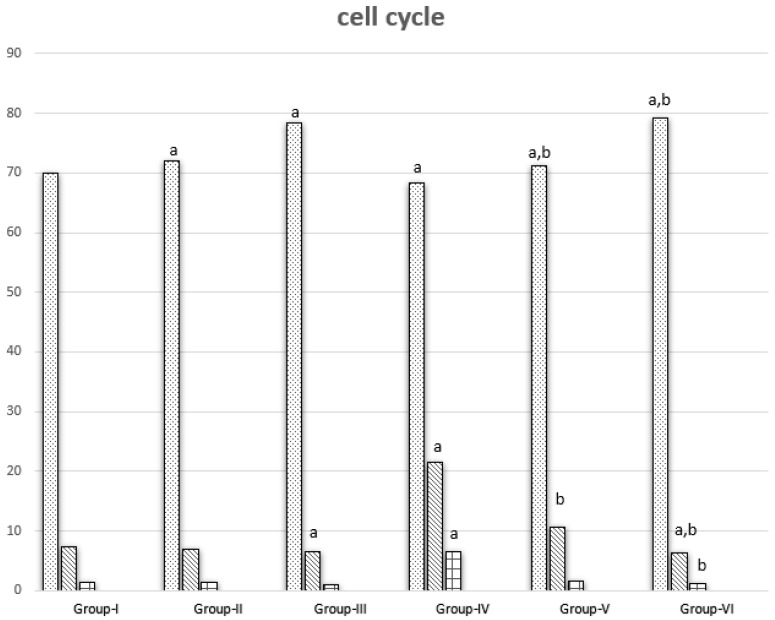
DMH, CUR, and CUR-loaded PLGA effect on cell cycle progression in various groups. a represents statically significant differences at (*p* < 0.05), compared different groups with Group-I and b represents statically significant differences at (*p* < 0.05), compared different groups with Group-IV.

**Figure 4:**
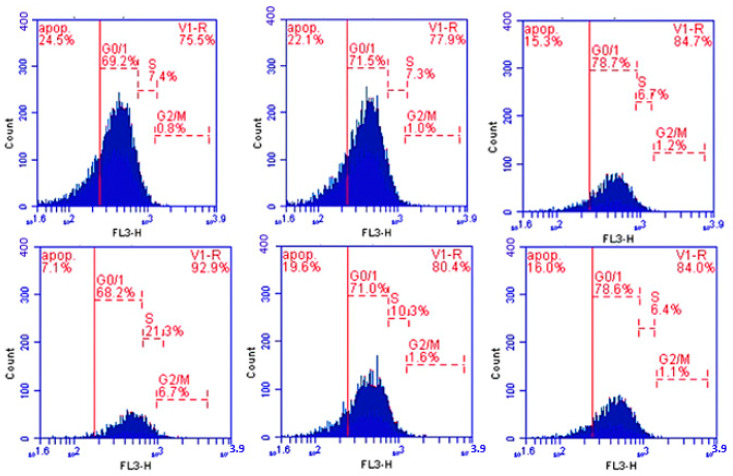
Histograms showing percentage (%) of cells in G0/1, S, and G2/M phases in different groups.

Cell cycle analysis was conducted in cells isolated from different groups. Groups II and III showed significant increase in G0\1 phase when compared with Group I. Group III showed a significant decrease in S phase when compared with Group I, while the same groups showed a nonsignificant change in G2\M phase. Group IV showed a significant decrease in G0\1 phase and a significant increase in S and G2\M phases compared with Group I. Groups V and VI showed a significant increase in G0\1 phase and a significant decrease in G2\M phase when compared with Group IV. Groups V and VI showed a non-significant and a significant decrease, respectively, in S phase. Group VI showed arrest of G0\1 phase (Figure 3 and Figure 4).

### 3.4. Histology of the Colon

**Figure 5:**
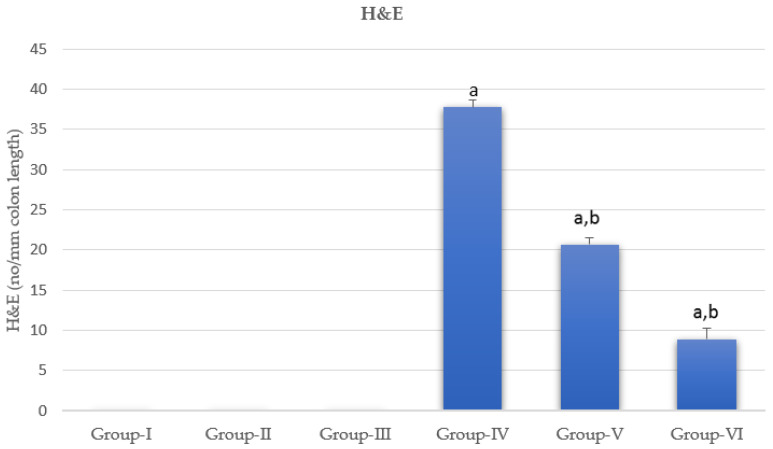
Quantitative assessment of dysplastic foci within different groups. a represents statically significant differences at (*p* < 0.05), compared different groups with Group-I and b represents statically significant differences at (*p* < 0.05), compared different groups with Group-IV.

**Figure 6:**
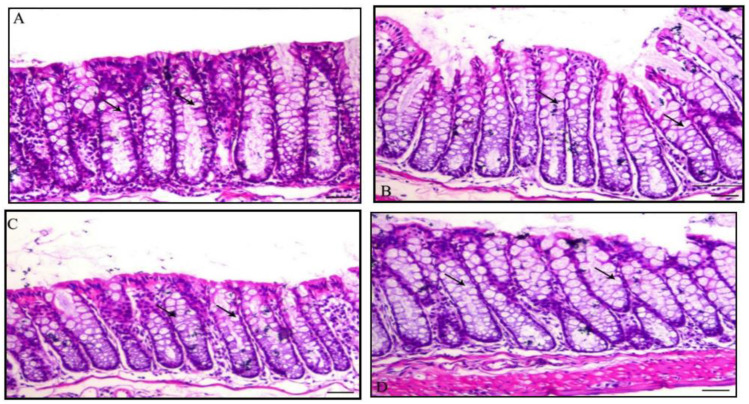
Photomicrographs of a normal colon section of mice stained with hematoxylin and eosin; (**A**): Colon of Group I animal showing normal intestinal crypts lined with columnar epithelium rich with goblet cells (arrows), H&E, ×200, bar = 50 μm; (**B**,**C**): Colon section of mice in Group II showing normal intestinal glands (arrows), H&E, ×200, bar = 50 μm; (**D**): Colon section of mice in Group III showing normal intestinal crypts (arrows), H&E, ×200, bar = 50 μm.

**Figure 7:**
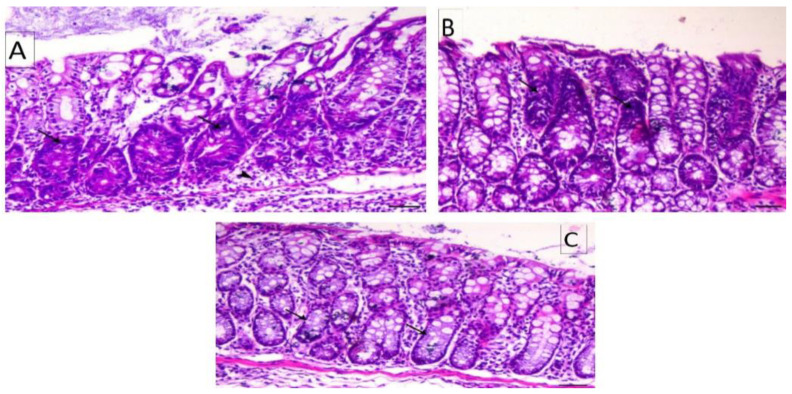
Photomicrographs of colon section of mice stained with hematoxylin and eosin; (**A**): Section from colon of Group IV positive animal treated with DMH showing marked dysplasia of the intestinal crypts (arrows) and interstitial colitis associated with inflammatory cells infiltration, mostly lymphocytes and macrophages (arrowhead), H&E, ×200, bar = 50 μm; (**B**): Section from colon of Group V showing decrease in both the dysplastic changes within the intestinal crypts (arrows) and interstitial inflammatory cells infiltration, H&E, ×200, bar = 50 μm; (**C**): Section from colon of Group VI showing marked decrease of intestinal dysplasia changes (arrows indicate dysplastic glands), H&E, ×200, bar = 50 μm.

Histological structure and the quantitative assessment of dysplastic foci of the mice colons from Group I and DMH-treated groups showed change. These changes were showed quantitatively in Figure 5. The normal colon wall is composed of an external serosal area of squamous simple epithelium followed by muscularis area, then with a lamina propria of connective tissues as shown in Figure 6 and Figure 7. The innermost layer, the mucosa, is formed of long mucosal crypts, which are branched tubular glands for mucin secretion. The mucosa from mice administered DMH were histologically distinguished by paler eosinophilia, slight dysplastic colonic crypts with wider openings, and notably fewer numbers of mucous-secreting cells. Parts of hyperplasia could be distinguished occasionally, and higher numbers of lymphocytic cells were frequently observed among the crypts. Quantitative assessments of dysplastic foci within different groups are represented in Figure 5.

### 3.5. Proliferating Cell Nuclear Antigen

**Figure 8:**
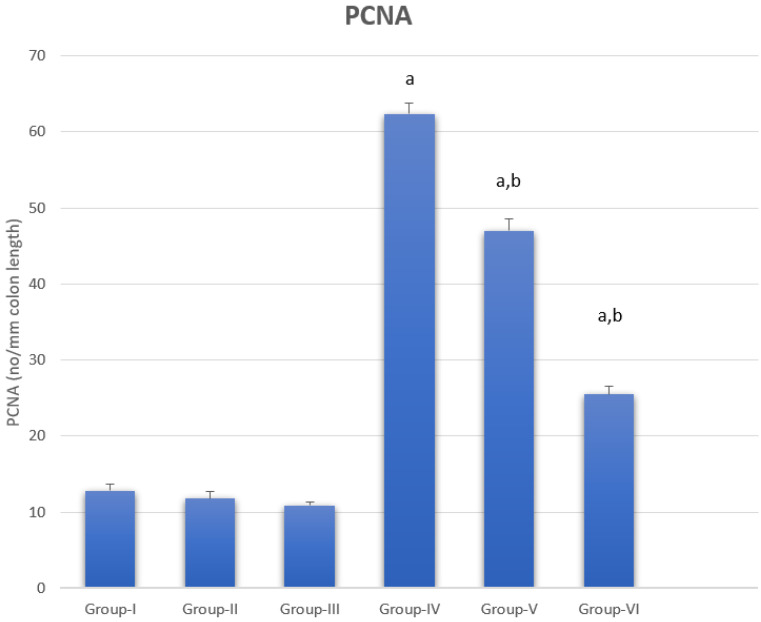
Proliferating cell nuclear antigen (PCNA) labelling index within different groups. a represents statically significant differences at (*p* < 0.05), compared different groups with Group-I and b represents statically significant differences at (*p* < 0.05), compared different groups with Group-IV.

**Figure 9:**
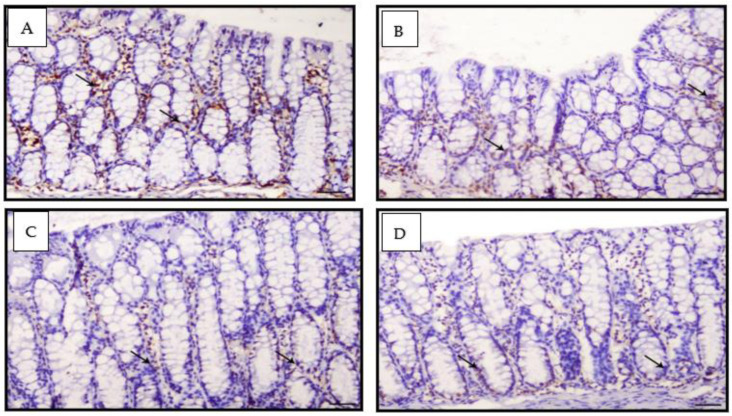
Photomicrographs of colon sections of mice stained IHC with PCNA; (**A**): Colon section of Group I animals showing mild expression of PCNA within the intestinal crypts (arrows), PCNA IHC, ×200, bar = 50 μm; (**B**,**C**): Colon section from Group II animals showing mild expression of PCNA within the intestinal crypts (arrows), PCNA IH, ×200, bar = 50 μm; (**D**) Colon section from Group III animals showing mild expression of PCNA within the intestinal crypts (arrows), PCNA IHC, ×200, bar = 50 μm.

**Figure 10:**
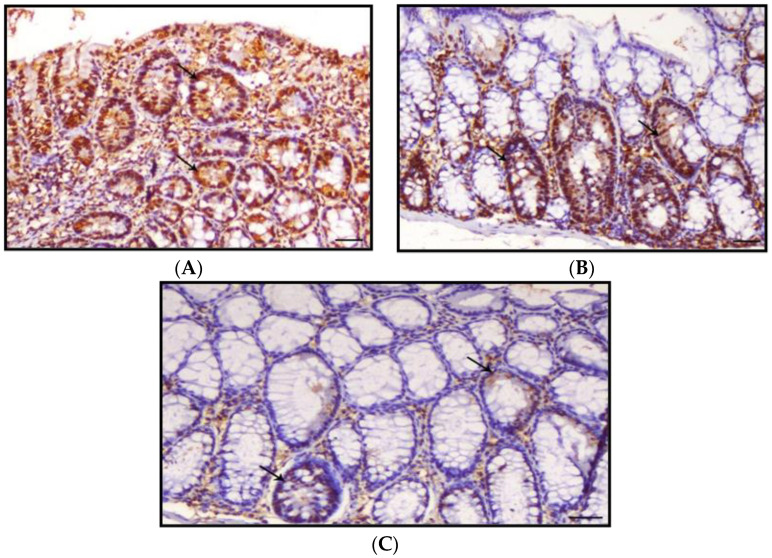
Photomicrographs of colon sections of mice stained IHC with PCNA; (**A**): Colon section of Group IV animals showing marked expression of PCNA within the intestinal crypts (arrows), PCNA IHC, ×200, bar = 50 μm; (**B**): Colon section of Group V animals showing a slight decrease in expression of PCNA within the intestinal crypts (arrows), PCNA IHC, ×200, bar = 50 μm; (**C**): Colon section of Group VI animals showing marked decrease in the expression of PCNA within the intestinal. Crypts (arrows), PCNA IHC, ×200, bar = 50 μm.

PCNA data as a sum of the different colonic regions of all groups are represented in Figure 8. The PCNA-LI positive nuclei were brownish in color, while non-stained nuclei were bluish. The proliferating cells were mainly restricted in the lower one-third of the colonic crypts of non-treated animals, as shown in Figure 9. The DMH-treated group showed a generally increased length of the proliferating zone in the colonic crypts to the middle and upper thirds (Figure 10). Generally, the LI was significantly higher in the colons of all DMH-treated mice. It was observed that PCNA-LI was significantly less in DMH+CUR and DMH+ CUR-loaded PLGA groups that showed a significant reduction in PCNA-positive cells as compared with the DMH-treated group.

## 4. Discussion

Phytochemicals such as CUR have a huge role in cancer treatment. Cancer cells acquire drug resistance by stimulating the secretion of anti-apoptotic proteins that stop the apoptosis in cancer cells, stimulation DNA repair. Chemotherapy induces DNA damage and stimulates apoptosis in cancer cells that already have resistance to these mechanisms. CUR as a phytochemical has the ability to initiate apoptosis by reducing anti-apoptotic protein secretion and activating P53 protein that stimulates apoptosis [4].

Nucleotide factor κB is a protein binding with DNA and stimulating transcription. This factor is activated by tumor necrosis factor alpha. The major role of phytochemicals, including CUR, is reducing TNF levels to stop DNA transcription [34].

Chemoprevention is the use of harmless plant-based elements, such as various dietary ingredients, to protect, prevent, stop, or reverse cancer growth. Lycopene, soy isoflavones, pomegranate phenolics, selenium, and curcumin (CUR), among other antioxidants, are present naturally in fruits and vegetables and may be employed as chemoprotective agents against carcinogenesis [35]. CUR nanoparticles boosted peroral bioavailability by at least nine times when compared to conventional CUR, according to studies [36]. In rats, a nano-formulation of CUR made of poly lactic-co-glycolic acid (PLGA) had a 22-fold higher oral bioavailability than conventional CUR [37].

In this study, when compared with the control group, DMH treatment resulted in a significant rise in TNF-alpha levels, which is consistent with the findings that DMH use may produce excessive ROS generation, which leads to TNF-alpha activation via the p65-NF-B pathway [38]. Through the generation of tumor necrosis factor-alpha (TNF-α), chronic inflammation promotes the transition of epithelial cells into cancer cells [39].

In this study, pro-inflammatory cytokines such as TNF-α, IL-1, and IL-6 were found to be lower in the DMH+CUR and DMH+CUR-loaded PLGA groups than in the DMH group. TNF- α, IL-1, and IL-6 are proinflammatory cytokines that are modulated by CUR [40]. Nano CUR, as phytochemicals, can have a significant anti-inflammatory effect, which is important for impeding tumor growth [41]. In investigations, CUR and the nanoCUR have been shown to suppress carcinogenesis in colorectal, gastric, pancreatic, hepatic, prostate, breast, oral malignancies and leukemia. Free radical scavenging activities of CUR, as well as a decrease in the expression of inflammatory cytokines IL-1b, IL-6, and TNF- α, result in decreased cancer growth and down-regulation of enzymes such as protein kinase C, which regulate inflammation and tumor cell proliferation [42].

In the current study, the usage of DMH resulted in a significant increase in vascular endothelial growth factor (VEGF) levels. The authors of [43] came to the same conclusion, interpreting it as signaling from the interaction between VEGF and its receptor, VEGFR, encouraging tumor formation and progression via endothelial cell invasion, migration, proliferation, and activation. As a result of this interaction, microvascular permeability was also increased [44,45].

In an in vitro endometriosis model, CUR suppresses VEGF suppression by downregulating VEGF expression and preventing hypoxia-induced angiogenesis [46]. VEGF was similarly suppressed by liposome CUR and CUR nanoparticles, which showed an antiangiogenesis effect [47]. Despite the fact that the conventional CUR group had much lower levels of VEGF in the current study, CUR encapsulated PLGA (NP) inhibited the expression of VEGF gene products more effectively than CUR alone, which is in line with prior studies [48]. The nanoCUR group had the least quantity of VEGF, according to the current findings, and hence our findings are consistent with [48,49]. CUR has been described in a number of publications as a natural anticancer extract capable of inducing cell cycle arrest. Cell cycle arrest was produced by CUR in the G0/G1 or G2/M stages [11]. We noticed that CUR caused cells to enter the G0/G1 phase in this investigation, which is in line with [50]. Both free and PLGA nanoCUR successfully reduced PCNA overexpression, with nanoCUR providing a substantially higher reduction than free CUR, as reported in [51]. Our data suggest that PCNA plays a key role in CUR-induced cell proliferation suppression, and that CUR may cause G1/S cell arrest in part by reducing PCNA expression, similar to how CUR causes G1/S cell arrest [52].

## 5. Conclusions

**Figure 11:**
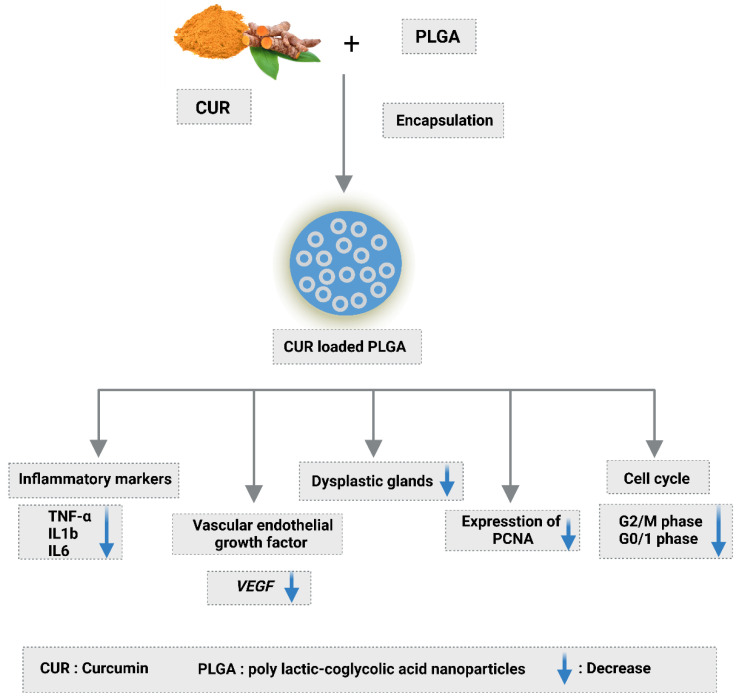
Nanocurcumin loaded PLGA effects.

From our study we concluded that nanocurcumin (Figure 11):

(1) Decreased G0/I phase in cell cycle;
(2) Lowered inflammatory markers and VEGF levels;
(3) Reduced intestinal crypt dysplasia and interstitial colitis linked with inflammatory cell infiltration, especially lymphocytes and macrophages;
(4) PCNA expression was improved by CUR-loaded PLGA;
(5) Curcumin’s bioavailability is severely reduced due to low serum levels, limited tissue distribution, apparent quick metabolism, and a short half-life. Longer circulation lengths, better permeability, and resistance to metabolic presystemic degradation were found in CUR @PLGA results;
(6) Curcumin’s activity becomes more potent after being placed onto nanoparticles.

## Data Availability

The data presented in this study are available on request from the corresponding author.
